# Laparoscopic Hepatectomy for Hepatic Inflammatory Myofibroblastic Tumor: A Case Report

**DOI:** 10.70352/scrj.cr.26-0104

**Published:** 2026-06-03

**Authors:** Yuta Hiura, Tomoyuki Abe, Megumi Yamaguchi, Michinori Hamaoka, Tsuyoshi Kobayashi, Hideki Ohdan, Kazuhiro Toyota

**Affiliations:** 1 Department of Gastroenterological Surgery, National Hospital Organization (NHO) Higashihiroshima Medical Center, Higashihiroshima, Hiroshima, Japan; 2 Department of Gastroenterological and Transplant Surgery, Graduate School of Biomedical and Health Sciences, Hiroshima University, Hiroshima, Hiroshima, Japan

**Keywords:** anaplastic lymphoma kinase-positive tumor, hepatic inflammatory myofibroblastic tumor, laparoscopic hepatectomy

## Abstract

**INTRODUCTION:**

Inflammatory myofibroblastic tumors (IMTs) are neoplasms composed of spindle-shaped myofibroblasts along with inflammatory infiltrate, and possess malignant potential. Primary hepatic IMTs (HIMTs) are relatively uncommon and difficult to distinguish from liver malignancies such as intrahepatic cholangiocarcinoma (ICC) and hepatocellular carcinoma due to the lack of characteristic radiological findings.

**CASE PRESENTATION:**

We present the case of a 76-year-old man with a medical history of renal cell carcinoma and gastric cancer. During routine follow-up, a CT revealed an irregular tumor measuring 30 mm in liver segment 5 (S5), which enlarged to 35 mm over a 6-month observation period. MRI demonstrated high signal intensity on diffusion-weighted imaging, while PET showed high standard uptake volume (11.2), suggestive of ICC or HIMT. A laparoscopic hepatic S5 sub-segmentectomy was performed. Pathological examination revealed spindle-cell proliferation with marked lymphoplasmacytic infiltration. The immunohistochemistry results were positive for anaplastic lymphoma kinase and smooth muscle actin. The patient was discharged on POD 10 and has remained recurrence-free 2 years following surgery.

**CONCLUSIONS:**

HIMT is rare and poses significant diagnostic challenges that often mimic other liver malignancies. This case report highlights the efficacy and safety of laparoscopic hepatectomy as both a diagnostic and therapeutic strategies.

## Abbreviations

ALK
: anaplastic lymphoma kinase
CD
: cluster of differentiation
c-kit
: cluster of differentiation 117
DWI
: diffusion-weighted imaging
EOB-MRI
: gadolinium-ethoxybenzyl-diethylenetriamine pentaacetic acid-enhanced MRI
FDG
: fluorodeoxyglucose
FISH
: fluorescence *in situ* hybridization
HIMT
: hepatic inflammatory myofibroblastic tumor
ICC
: intrahepatic cholangiocarcinoma
IgG
: immunoglobulin G
IMT
: inflammatory myofibroblastic tumor
IPT
: inflammatory pseudotumor
NSAIDs
: nonsteroidal anti-inflammatory drugs
RET
: rearranged during transfection proto-oncogene
ROS1
: receptor tyrosine kinase
S5
: liver segment 5
SMA
: smooth muscle actin
SUVmax
: maximum standardized uptake value

## INTRODUCTION

IMTs are unique mesenchymal neoplasms characterized by the proliferation of myofibroblastic spindle cells and a significant inflammatory response involving lymphocytes, plasma cells, and eosinophils. Historically, this disease has been classified under the umbrella term “IPT,” which refers to a broad group of nonneoplastic inflammatory mass-forming lesions. However, owing to its tendency to recur locally and the risk for distant metastasis, the World Health Organization has reclassified IMT as a borderline tumor with intermediate biological potential.^1)^ IMTs most frequently arise in the lung and abdominal cavity, particularly in the mesentery and omentum, as well as in the retroperitoneum. They can also occur at various other sites, including the gastrointestinal tract, pelvis, mediastinum, genitourinary tract, and head and neck; however, a hepatic origin is rare.^2)^ Although the precise incidence among patients undergoing hepatic tumor resection remains unclear, previous reports have suggested that hepatic IPTs, in a broad sense, account for approximately 0.7% of all resections of localized liver lesions.^3)^ The most common clinical symptom is abdominal pain (53%), followed by fever (41%), and 15.6% of patients are asymptomatic.^4)^ IMTs are typically not associated with elevated tumor markers and lack characteristic imaging findings, making their diagnosis challenging.^5)^ Herein, we present a case of ALK-positive HIMT that mimicked ICC, which was successfully treated with laparoscopic sub-segmentectomy.

## CASE PRESENTATION

We present the case of a 76-year-old man who was being followed up at our hospital for early gastric cancer, whose annual CT detected an irregularly shaped tumor measuring 30 mm in S5. His previous medical history included a retroperitoneoscopic left nephrectomy for left kidney cancer 7 years earlier, a partial retroperitoneoscopic right nephrectomy for right metachronous renal cancer 6 years earlier, and an endoscopic submucosal dissection for early gastric cancer 4 years earlier. The patient’s height, weight, and BMI were 166.5 cm, 60.4 kg, and 21.5 kg/m^2^, respectively. Laboratory findings were as follows: a white blood cell count of 7100/μL, an alpha-fetoprotein level of 33.4 ng/mL, a carcinoembryonic antigen level of 6.9 ng/mL, and a carbohydrate antigen 19-9 level of 2.8 IU/mL.

**Fig. 1:**
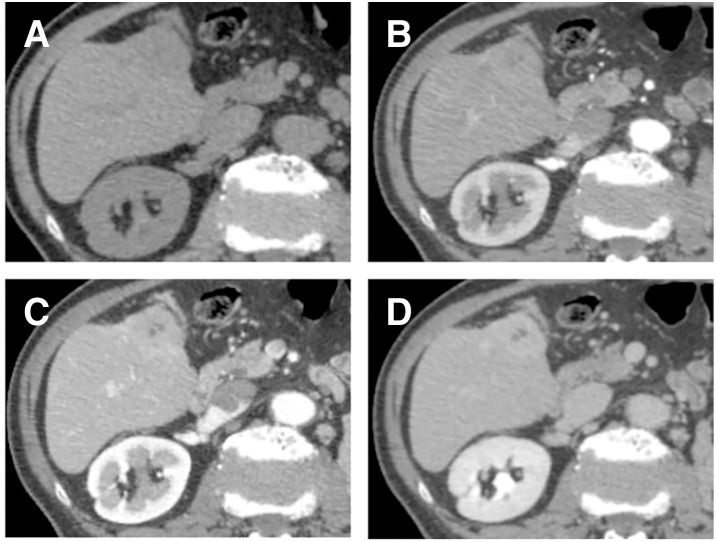
Contrast-enhanced CT imaging of the liver. (**A**) Plain CT showing a 35-mm mass in segment 5 of the liver. (**B**) Arterial-phase contrast-enhanced CT showing mild heterogeneous enhancement of the hepatic lesion. (**C**) Portal venous phase showing gradual progression of the intralesional enhancement, demonstrating a progressive (delayed) enhancement pattern. (**D**) Delayed enhancement of the lesion in equilibrium-phase CT.

**Fig. 2:**
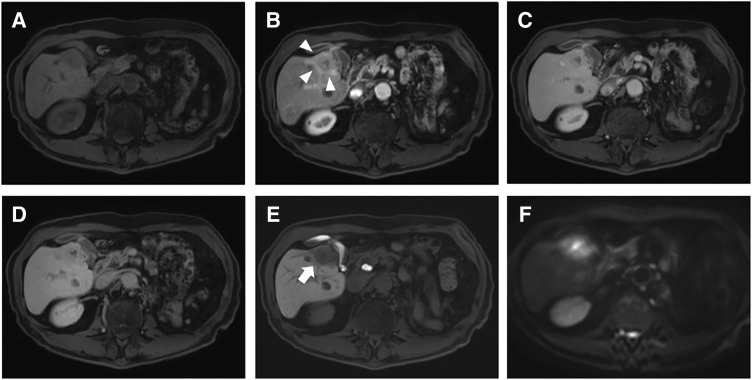
(**A**) The unenhanced image shows a 38-mm hypointense lesion in segment 5 of the liver. (**B**) Arterial-phase MRI demonstrates peritumoral enhancement (white arrowheads) with multifocal necrotic areas in the lesion. (**C**) Portal venous-phase image shows faint progressive internal enhancement in the lesion. (**D**) Transitional-phase image shows faint enhancement in the lesion. (**E**) During the 20-min hepatobiliary phase after contrast administration, a peritumoral hypointense area is observed (white arrow). (**F**) DWI shows abnormal signal intensity of the lesion. DWI, diffusion-weighted imaging

**Fig. 3:**
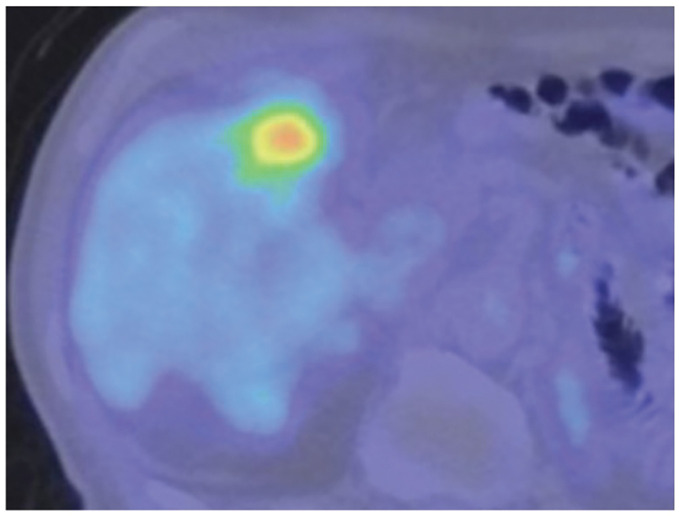
PET-CT imaging showing markedly increased tracer uptake by the lesion.

The hepatic tumor was irregular in shape and showed poor enhancement on CT imaging. MRI revealed a wedge-shaped tumor with a low signal on T1WI, mixed high signal intensity on T2WI, and an abnormal DWI signal. It was not typical of hepatocellular carcinoma (HCC) or ICC, and a metastatic tumor was considered unlikely. Surgical resection for diagnostic and therapeutic purposes was considered; however, follow-up observation was chosen in accordance with the patient’s strong preference. Contrast-enhanced CT performed at 6-month follow-up showed that the tumor had increased in size to 35 mm (**Fig. 1**). MRI DWI revealed dominant areas of high signal intensity within the tumor (**Fig. 2**). PET-CT showed hyperintensity with a SUVmax of 11.2 only in this localized area (**Fig. 3**).

**Fig. 4:**
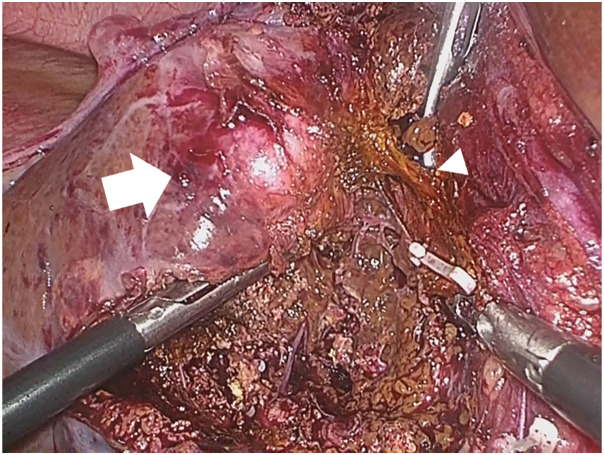
On intraoperative gross examination, a firm, gray-white mass was observed (white arrow), and Glisson’s sheath 5 was dissected and divided (white arrowhead).

Owing to the suspicion of ICC or HIMT, a laparoscopic hepatic S5 sub-segmentectomy was performed to ensure a sufficient surgical margin (R0 resection) (**Fig. 4**). The operation time was 304 min, and the intraoperative bleeding volume was 10 mL. The postoperative course was uneventful, and the patient was discharged on POD 10.

**Fig. 5:**
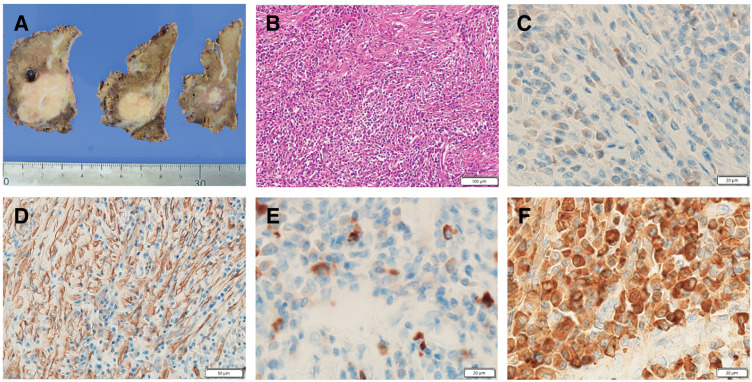
Histopathological and immunohistochemical findings. (**A**) The tumor is well circumscribed, showing a yellow-white solid appearance on the cut surface. (**B**) Hematoxylin–eosin staining of the lesion shows predominantly fascicular proliferation of spindle cells with mild atypia, accompanied by dense infiltration of lymphocytes and plasma cells. (**C**) Immunohistochemistry shows positive ALK-1 expression. (**D**) Immunohistochemistry shows positivity for SMA. (**E**) IgG4-positive cells are visible within the tumor (63 per high-power field). (**F**) The IgG4/IgG ratio is approximately 20%. ALK, anaplastic lymphoma kinase; IgG, immunoglobulin G; SMA, smooth muscle actin

Pathological examination of the resected specimen revealed a lesion composed primarily of bundles of spindle-shaped cells with mild atypia, intermingled with numerous lymphocytes, plasma cells, histiocytes, and fibroblasts. Immunohistochemistry showed expression of ALK-1 and alpha-SMA, but not CD34, CD117 (c-kit), desmin, calcium-binding protein (S100), or epithelial membrane antigen. We observed 63 IgG4-positive cells per high-power field, and the IgG4/IgG ratio was approximately 20% (**Fig. 5**). Therefore, a postoperative diagnosis of HIMT was established. The patient has remained recurrence-free for 2 years postoperatively.

## DISCUSSION

IMT was previously regarded as a reactive IPT secondary to surgery, trauma, or infection.^6)^ However, the subsequent identification of recurrent rearrangements involving ALK, as well as fusion of other ROS1 genes, such as ROS proto-oncogene 1, ROS1, platelet-derived growth factor receptor beta, and RET, has established IMT as a clonal intermediate-grade neoplasm driven by these kinase fusions.^7)^ Chang et al. identified kinase fusions in all 33 analyzed cases of thoracic IMT, with ALK rearrangements being the most common and with approximately 30% of cases involving other kinases, such as ROS1, NTRK3, and RET.^8)^ No clear association has been established between specific fusion genes and the background liver in hepatic IMT. However, recent studies suggest that molecular alterations may correlate with morphological features: ALK-positive tumors tend to be hypercellular with diffuse SMA expression, whereas ETV6:NTRK3-rearranged tumors may show hypocellular morphology with myxoid or collagenous stroma.^9)^ In the present case, immunohistochemistry confirmed ALK positivity, supporting the neoplastic nature of the lesion despite the unknown cause.

Several reports have indicated that HIMT can mimic primary hepatic malignancies, including HCC and ICC, as well as metastatic liver tumors arising from gastrointestinal cancers; therefore, complete surgical resection is considered an appropriate management strategy from both diagnostic and therapeutic standpoints.^10,11)^ Although discrimination based on imaging findings alone is challenging, CT typically demonstrates delayed enhancement associated with fibrosis and heterogeneous enhancement associated with central necrosis.^12)^ However, ICC can exhibit a similar enhancement pattern, and differentiation was challenging in our case as well. On EOB-MRI, IMT frequently demonstrates peritumoral hyperintensity in the arterial phase and peritumoral hypointensity in the hepatobiliary phase. In addition, rapid tumor regression on follow-up imaging may be a useful feature for differentiating IMT from other malignant hepatic lesions.^13)^ Moreover, Chang et al. reported that IMT and ICC exhibit peritumoral hyperintensity at slightly different time points: IMT tends to show this finding during the early dynamic phases, whereas ICC more often demonstrates delayed peritumoral hyperintensity in the transitional and hepatobiliary phases, along with a similar pattern on DWI.^14)^ In our case, a similar enhancement pattern was observed, and HIMT was suspected preoperatively. However, the tumor did not show regression after 6 months of observation; therefore, surgical resection was performed for both diagnostic and therapeutic purposes. EOB-MRI is essential for precise preoperative diagnosis of HIMT. As additional imaging findings, in most cases, PET-CT reveals markedly increased ^18^F-FDG uptake, which may lead to the misdiagnosis of malignant tumors. Previous studies have reported that higher tumor cell density, greater inflammatory cell infiltration, and increased Ki-67 expression correlate with higher SUVmax values, suggesting a possible relationship between these histopathological features and ^18^F-FDG accumulation.^15)^

The diagnosis of IMT is made based on histopathological examination. When HIMT is suspected, tumor biopsy is an alternative approach to make a precise diagnosis and avoid surgery.

Nevertheless, several previous reports have described surgical resection even after a biopsy-based diagnosis of hepatic IMT, likely reflecting the intermediate biological behavior of IMT and the difficulty in excluding malignancy completely.^16,17)^ Furthermore, hepatic epithelioid hemangioendothelioma has been reported to be misdiagnosed as hepatic IMT after liver biopsy, indicating that differential diagnosis may be challenging in some cases.^18)^ Jacob et al. described a systemic IMT involving the liver in which liver biopsy, together with ALK immunohistochemistry and confirmatory FISH for ALK rearrangement, enabled a molecularly supported diagnosis.^19)^ Their report highlights the potential utility of FISH as an adjunctive diagnostic tool when IMT is suspected on biopsy material, particularly in cases where nonsurgical management or targeted therapy may be considered. Considering the risk of needle tract seeding if the tumor had been malignant and insufficient sample volume, we opted for diagnostic and therapeutic resection. IMTs typically show strong immunoreactivity for SMA and vimentin, while desmin and cytokeratin may be focally positive. In addition, ALK positivity is observed in approximately 40%–70% of cases, with staining patterns varying according to the fusion partner. Conversely, markers such as S100, myogenin, c-kit, and β-catenin are usually negative, which is useful for distinguishing IMT from other soft tissue tumors or sarcomas.^20)^ A critical differential diagnosis of HIMT is IgG4-related sclerosing disease. IMT can be distinguished from IgG4-related liver disease by the presence of ALK expression, lower infiltration of IgG4-positive plasma cells, and the absence of obliterative phlebitis.^5)^ ALK positivity has been associated with local recurrence but not with distant metastasis, and is generally considered a favorable prognostic indicator.^21)^

The optimal treatment strategy for IMT depends on its resectability and molecular status. Durable partial responses to ALK inhibitors have been reported.^22)^ Even in ALK-negative IMT, where ALK inhibition is not a therapeutic option, NSAIDs may induce complete remission. Therefore, NSAIDs should be considered for patients with unresectable or recurrent IMT.^23)^ Treatment of IMT of the cavernous sinus and skull base generally involves a combination of total resection and steroid therapy, and steroid use may inhibit disease progression.^24)^ Moreover, radiotherapy is effective for IMTs arising in the nasopharynx and skull.^25)^ However, in HIMT, case reports of remission achieved with nonsurgical treatment alone are exceedingly scarce. Among 64 patients with HIMTs reported by Tang et al., all patients underwent hepatic resection, with no recurrences observed during a median follow-up of 30 months, leading the authors to conclude that complete resection can provide favorable long-term outcomes in HIMT.^4)^ Furthermore, recent case reports on HIMT have documented numerous patients who remained recurrence-free for several years after R0 resection.^26–29)^ Therefore, complete surgical resection is currently considered the first-line treatment and gold standard for HIMT.

## CONCLUSIONS

HIMT is a rare entity that often mimics malignant liver tumors on imaging, making preoperative diagnosis challenging. This case demonstrates that when malignancy cannot be reliably excluded, complete surgical resection provides both definitive diagnosis and effective treatment. The favorable long-term outcome observed supports hepatectomy as the standard management strategy for suspected HIMT and highlights its importance in guiding clinical decision-making for indeterminate hepatic lesions.

## DECLARATIONS

### Availability of data and materials

Data sharing is not applicable to this article as no datasets were generated or analyzed in the current study.

## References

[1] WHO Classification of Tumours Editorial Board. WHO classification of tumours. Vol. 3: Soft Tissue and Bone Tumours. 5th ed. Lyon: IARC Publications; 2013.

[2] Choi JH. Inflammatory myofibroblastic tumor: an updated review. Cancers (Basel) 2025; 17: 1327. 40282503 10.3390/cancers17081327 PMC12026078

[3] Torzilli G, Inoue K, Midorikawa Y, et al. Inflammatory pseudotumors of the liver: prevalence and clinical impact in surgical patients. Hepatogastroenterology 2001; 48: 1118–23. 11490814

[4] Tang L, Lai EC, Cong WM, et al. Inflammatory myofibroblastic tumor of the liver: a cohort study. World J Surg 2010; 34: 309–13. 20033408 10.1007/s00268-009-0330-x

[5] Elpek GÖ. Inflammatory myofibroblastic tumor of the liver: A diagnostic challenge. J Clin Transl Hepatol 2014; 2: 53–7. 26356188 10.14218/JCTH.2013.00023 PMC4521256

[6] Ryu KH, Im CM, Kim MK, et al. Inflammatory myofibroblastic tumor of the kidney misdiagnosed as renal cell carcinoma. J Korean Med Sci 2010; 25: 330–2. 20119595 10.3346/jkms.2010.25.2.330 PMC2811309

[7] Lovly CM, Gupta A, Lipson D, et al. Inflammatory myofibroblastic tumors harbor multiple potentially actionable kinase fusions. Cancer Discov 2014; 4: 889–95. 24875859 10.1158/2159-8290.CD-14-0377 PMC4125481

[8] Chang JC, Zhang L, Drilon AE, et al. Expanding the molecular characterization of thoracic inflammatory myofibroblastic tumors beyond ALK gene rearrangements. J Thorac Oncol 2019; 14: 825–34. 30550870 10.1016/j.jtho.2018.12.003 PMC6486847

[9] Han Q, Zhang Z, He X, et al. Primary inflammatory myofibroblastic tumour of the liver: a clinicopathological and genetic study including a subset with ETV6: NTRK3 fusion. Histopathology 2023; 82: 925–36. 36748182 10.1111/his.14881

[10] Venkataraman S, Semelka RC, Braga L, et al. Inflammatory myofibroblastic tumor of the hepatobiliary system: report of MR imaging appearance in four patients. Radiology 2003; 227: 758–63. 12728186 10.1148/radiol.2273020572

[11] Liu XF, He BM, Ou-Yang XH, et al. Different imaging findings of inflammatory myofibroblastic tumor of the liver. World J Gastroenterol 2012; 18: 5821–5. 23155327 10.3748/wjg.v18.i40.5821 PMC3484355

[12] Surabhi VR, Chua S, Patel RP, et al. Inflammatory myofibroblastic tumors: current update. Radiol Clin North Am 2016; 54: 553–63. 27153788 10.1016/j.rcl.2015.12.005

[13] Kang TW, Kim SH, Jang KM, et al. Inflammatory myofibroblastic tumours of the liver: gadoxetic acid-enhanced and diffusion-weighted MRI findings with 18F-FDG PET/CT and clinical significance of regression on follow-up. Clin Radiol 2014; 69: 509–18. 24581965 10.1016/j.crad.2013.12.018

[14] Chang AI, Kim YK, Min JH, et al. Differentiation between inflammatory myofibroblastic tumor and cholangiocarcinoma manifesting as target appearance on gadoxetic acid-enhanced MRI. Abdom Radiol (NY) 2019; 44: 1395–406. 30515535 10.1007/s00261-018-1847-y

[15] Dong A, Wang Y, Dong H, et al. Inflammatory myofibroblastic tumor: FDG PET/CT findings with pathologic correlation. Clin Nucl Med 2014; 39: 113–21. 23797227 10.1097/RLU.0b013e3182952caa

[16] Yang S, Tang Y, Yuan Z, et al. Inflammatory myofibroblastic tumor in the liver after bone marrow transplantation: case report and literature review. Front Med (Lausanne) 2025; 12: 1489399. 40224630 10.3389/fmed.2025.1489399 PMC11986994

[17] Kruth J, Michaely H, Trunk M, et al. A rare case of fever of unknown origin: inflammatory myofibroblastic tumor of the liver. Case report and review of the literature. Acta Gastroenterol Belg 2012; 75: 448–53. 23402091

[18] Zhao J, Dang Y. Case report of misdiagnosis: a rare case of hepatic epithelioid hemangioendothelioma characterized primarily by fever. Front Oncol 2025; 15: 1606872. 40958872 10.3389/fonc.2025.1606872 PMC12434749

[19] Jacob SV, Reith JD, Kojima AY, et al. An unusual case of systemic inflammatory myofibroblastic tumor with successful treatment with ALK-inhibitor. Case Rep Pathol 2014; 2014: 470340. 25045570 10.1155/2014/470340 PMC4087275

[20] Chmiel P, Słowikowska A, Banaszek Ł, et al. Inflammatory myofibroblastic tumor from molecular diagnostics to current treatment. Oncol Res 2024; 32: 1141–62. 38948020 10.32604/or.2024.050350 PMC11209743

[21] Coffin CM, Hornick JL, Fletcher CD. Inflammatory myofibroblastic tumor: comparison of clinicopathologic, histologic, and immunohistochemical features including ALK expression in atypical and aggressive cases. Am J Surg Pathol 2007; 31: 509–20. 17414097 10.1097/01.pas.0000213393.57322.c7

[22] Tothova Z, Wagner AJ. Anaplastic lymphoma kinase-directed therapy in inflammatory myofibroblastic tumors. Curr Opin Oncol 2012; 24: 409–13. 22664824 10.1097/CCO.0b013e328354c155

[23] Chavez C, Hoffman MA. Complete remission of ALK-negative plasma cell granuloma (inflammatory myofibroblastic tumor) of the lung induced by celecoxib: a case report and review of the literature. Oncol Lett 2013; 5: 1672–6. 23761833 10.3892/ol.2013.1260 PMC3678867

[24] McCall T, Fassett DR, Lyons G, et al. Inflammatory pseudotumor of the cavernous sinus and skull base. Neurosurg Rev 2006; 29: 194–200. 16565875 10.1007/s10143-006-0017-9

[25] Gabel BC, Goolsby M, Hansen L, et al. Inflammatory myofibroblastic tumor of the left sphenoid and cavernous sinus successfully treated with partial resection and high dose radiotherapy: case report and review of the literature. Cureus 2015; 7: e328. 26543686 10.7759/cureus.328 PMC4627831

[26] Shen L, Yang Z, Ding R, et al. Hepatic inflammatory myofibroblastic tumor: one case report. Front Surg 2022; 9: 902753. 35990088 10.3389/fsurg.2022.902753 PMC9386116

[27] Meng Y, Xie J, Liang Y, et al. Inflammatory myofibroblastic tumor in the liver: a case report. Front Oncol 2024; 14: 1349692. 38863636 10.3389/fonc.2024.1349692 PMC11165177

[28] Huang K, Zhao P, Zhao J, et al. A unique case of inflammatory myofibroblastic tumor of the liver manifesting with biloma: a case report. Oncol Lett 2022; 24: 227. 35720485 10.3892/ol.2022.13348 PMC9185161

[29] Filips A, Maurer MH, Montani M, et al. Inflammatory myofibroblastic tumor of the liver: a case report and review of literature. World J Hepatol 2020; 12: 170–83. 32685109 10.4254/wjh.v12.i4.170 PMC7336290

